# Representing and comparing protein structures as paths in three-dimensional space

**DOI:** 10.1186/1471-2105-7-460

**Published:** 2006-10-20

**Authors:** Degui Zhi, S Sri Krishna, Haibo Cao, Pavel Pevzner, Adam Godzik

**Affiliations:** 1 Department of Plant and Microbial Biology, University of California, Berkeley, California 94720-3102, USA; 2 Joint Center for Structural Genomics, Burnham Institute for Medical Research, La Jolla, CA 92037, USA; 3 Bioinformatics Program, Infectious and Inflammation Disease Center, Burnham Institute for Medical Research, La Jolla, CA 92037, USA; 4 Department of Computer Science and Engineering, University of California, San Diego, La Jolla, California 92093-0114, USA

## Abstract

**Background:**

Most existing formulations of protein structure comparison are based on detailed atomic level descriptions of protein structures and bypass potential insights that arise from a higher-level abstraction.

**Results:**

We propose a structure comparison approach based on a simplified representation of proteins that describes its three-dimensional path by local curvature along the generalized backbone of the polypeptide. We have implemented a dynamic programming procedure that aligns curvatures of proteins by optimizing a defined sum turning angle deviation measure.

**Conclusion:**

Although our procedure does not directly optimize global structural similarity as measured by RMSD, our benchmarking results indicate that it can surprisingly well recover the structural similarity defined by structure classification databases and traditional structure alignment programs. In addition, our program can recognize similarities between structures with extensive conformation changes that are beyond the ability of traditional structure alignment programs. We demonstrate the applications of procedure to several contexts of structure comparison. An implementation of our procedure, CURVE, is available as a public webserver.

## Background

Knowledge of protein three-dimensional (3-D) structure is a prerequisite to understanding its function at a molecular level. With more than 37,000 protein structures in the rapidly growing public repository PDB [1], the importance of computer algorithms that can rapidly compare and find remote similarities between these structures cannot be over-emphasized. The comparison of protein structures has been an extremely important problem in structural and evolutionary biology ever since the first few protein structures became available. Hundreds of algorithms for protein structure comparison have been developed; there are several large databases and WEB resources devoted almost entirely to the problem of comparing and classifying protein structures, such as SCOP [2,3], CATH [4,5], and the DALI domain dictionary [6].

Typically, different representations of protein structure are employed for different contexts of structure comparisons. For example, an all-atom protein model is useful when studying finer details of a protein structure such as the subtle changes in the side-chain conformations of the active site residues upon substrate binding. However, for the rapid comparison of protein structures in order to find global similarities, only one point per residue, often the position of its C_α _atom, is generally sufficient. Some programs use completely different representations of protein structures, such as distance matrices [7], secondary structure vectors [8], or mesostates of backbone dihedral angles [9].

All protein structure alignment programs optimize some mathematical definition of structural similarity. The most popular measure of structural similarity is the root mean squared deviation (RMSD) of the aligned atoms [10] and its variants [11]. In general, alignments optimizing different measures of structural similarity may be different from each other [12]. Moreover, structural alignment is an NP-hard computational problem [13] and in order to solve it in a realistic time various heuristics have been developed, such as, lowering the dimensionality of the problem by identifying 7 × 7 residue interaction patterns in DALI [7], describing the protein as a set of vectors based on secondary structure elements in VAST [8], or using local structural similarities to identify short aligned fragment pairs (AFPs), which are used later to construct the alignment in methods such as CE [13] and FATCAT [14].

Since algorithms that optimize RMSD dominate the field of structure comparison, they create a misconception that only structures that can be superimposed with reasonable RMSD criteria, such as low RMSD over a large number of residues of the proteins, should be considered similar. While this is a pragmatic definition of structural similarity that eliminates an excess of false-positive matches, it fails to find similarities between structures with extensive conformation changes including structures with internal rearrangements and/or with swapped elements between domains. The recent years have seen advances in algorithms that can align protein structures assuming flexibility of their polypeptide chains [14,15]. Expert-curated structure classifications (such as SCOP and CATH) have dealt with this problem indirectly, by using highly abstracted, but not precisely defined, views of protein structure (fold) and by grouping together protein structures based on a combination of sequence, structural, functional, and evolutionary information. The rapid accumulation of new structures, however, outpaces the manual curation efforts, and automatic means of detecting structural similarities, which are beyond the scope of RMSD-based structure alignment programs, are becoming essential.

In this manuscript, we propose a very general abstraction of protein structure that views it as a path in 3-D space, and describe a novel dynamic programming algorithm for structure comparison by aligning the turning angle series and comparing our results with the structural similarity defined by the SCOP database. Surprisingly, even at this clearly oversimplified level of protein structure description, our benchmarking results are in a good agreement with the SCOP classification and existing structure alignment programs. Due to the flexibility encoded in our formulation, we demonstrate that our methods can find uses in assessing structure predictions, comparing structures with extensive distortion, modeling structure families, and revealing potential remote homology.

## Results

### Aligning angle series along smoothed backbone

Our approach for abstracting the protein structure is inspired by an earlier work from our group [16] and the U-turn model [17]. We developed a highly simplified description of protein structure that minimizes local structural information by "smoothing" the protein backbone, leaving only information about whether a protein chain is locally straight or curved. In particular, we "smooth" the protein backbone by averaging C_α _position in a seven-residue window [16]. Chain fragments that remain straight after the smoothing procedure are denoted as *generalized secondary structure *elements. Local secondary structural information is partially lost, and protein structure is abstracted to a path in 3-D space, which for a typical protein structure winds through space by following a straight line for a 5–12 residues, then turning in a typically 4–5 residue turn only to assume a straight course for another 5–12 residues.

**Figure 1:**
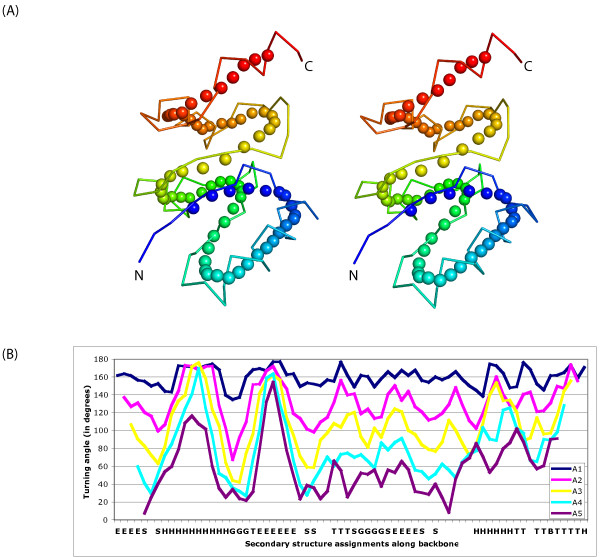
**Backbone smoothing and turning angle series of the structure with SCOP id d1b6ra2**. (A) Stereo images of overlapping backbone and smoothed backbone with smoothing radius d = 3; (B) turning angle series along the smoothed backbone with different angle defining distances, with X-axis labeled by DSSP [38] secondary structure annotation. Data series: A1, A2,..., A5: angle series with angle defining distance d = 1, 2,..., 5, respectively. All structural diagrams are prepared by using PyMOL [39].

We represent these characteristics by describing such paths as series of turning angles along the (generalized) backbone (Figure 1) (See Methods section for details). Intuitively, the angles are close to 180° along the straight fragments and are smaller where the backbone is changing directions. Turning angle series has several advantages for being a good descriptor of protein structures: (i) It is invariant to rotation and translation. (ii) It is tolerant to hinges, bending, or other structural distortions since these are reflected as small and well-localized changes of turning angles. (iii) By treating the one-dimensional (1-D) turning angle series as a sequence of numbers, one can define the problem of comparing structures as aligning the angle series, similar to traditional sequence alignment, for which an optimal solution can be derived by standard dynamic programming techniques [18,19].

The idea of 1-D geometric descriptions of structures and the dynamic programming alignment methods have been explored previously [20,21], including the use of curvature and torsion angles of the backbone to describe the local chain structure [22]. However, these methods differ significantly from our method at the level of structure abstraction in that they typically provide a much richer and more detailed description of the protein. Our method focuses only on turning angles in a generalized (smoothed) protein backbone, thus providing a somewhat minimal structure description. The idea of protein backbone smoothing was explored before in different contexts [23,24]. Interestingly, as we will show in this manuscript, in the world of natural protein structures, this minimal information encoded in our representation is often sufficient to recognize similarity between structures.

We have implemented a dynamic programming procedure as a computer program, CURVE, which compares structures by aligning the turning angles along their generalized backbones. Below we first present our benchmarking results, and then discuss applications of CURVE as applied to different contexts of structure comparison.

### The angle series alignment mostly agrees with existing measures of structural similarity

To evaluate the ability of CURVE in recognizing structural similarity, we first conducted a benchmark test. We compare CURVE against CTSS [22] and CE using the setting that was used by Can and Wang [22]. Specifically, 100 structures were chosen randomly from a database of 2939 representative structures and each of them was used as a query to search against the database. A hit was called if there was at least one match from the same superfamily in the top ranking list (top 1, 5, or 10). Table 1 compares our results with CE and CTSS. CE's performance is superior as it uses complete 3-D information. Our results are comparable to CTSS, which not only uses curvatures but also torsion angles and secondary structure information. Our results suggest that when using the curvature angle for structure comparison, curvature is the most important feature among the ones that are being used and the addition of other features such as torsion angles and secondary structure does not significantly improve the sensitivity of the algorithm.

**Table 1:** Benchmarking result of CURVE compared against CE and CTSS.

Method	Rank1	Rank5	Rank10
CE	88	90	92
CTSS	55	73	82
CURVE	61	67	82

In our second experiment, we take representative structural domains from three of the major SCOP classes, all-alpha, all-beta, and alpha-and-beta (a/b) proteins, and compare them against all structures in a 90% non-redundant set of SCOP version 1.65 (SCOP165_90) [25] which contained 8666 structures.

**Figure 2:**
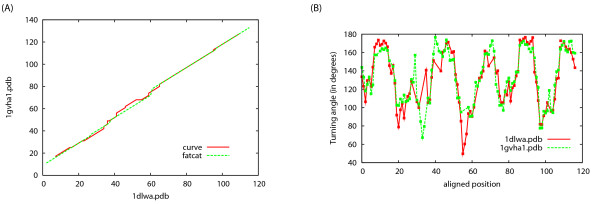
**Alignment of truncated hemoglobin 1dlw:a to globin 1ghv:a1**. (A) Dotplot of the alignments generated by FATCAT and CURVE; (B) angle curve overlap graph.

In the first example we search 1dlw:A, a truncated hemoglobin (SCOP classification a.1.1.1) against SCOP165_90. The top 5 hits include members of the same SCOP family (a.1.1.1), followed by domains from the globins family (a.1.1.2). Figure 2 shows the detailed CURVE alignment for 1dlw:A vs 1gvh:A1. As a comparison, the alignment dotplot generated by FATCAT[14] is overlaid with the alignment dotplot generated by CURVE in Figure 2A. The alignment paths of CURVE and FATCAT have an excellent overall agreement. Due to the smoothing procedure, the turning angle series is shorter at both termini, and the CURVE alignment path is correspondingly shorter.

Immunoglobulins are a large family of proteins with 404 structures represented in the SCOP165_90 set. Our second example is a search with one of the immunoglobulin structures, 1clo:h1. CURVE was able to identify 376 immunoglobulins among the top 400 hits. 23 of those 24 false positives belong to the all-beta class; the only non-all-beta hit belongs to the a+b class.

Arguably, this is a result of our simplistic affine gap penalty scheme. Most of the false positives have a larger number of residues in gaps. In fact, if one requires that number of residues in gaps to be less than 30, the top 400 hits contain only 9 non-immunoglobulin hits and all of them are from the all-beta class.

Our third search is with 1b7b:A, which is a relatively small a/b protein, from the Carbamate kinase-like fold. CURVE scores all structures from this fold higher than structures from other folds.

In summary, we found that although CURVE uses very limited structural information, its performance is comparable to CTSS (another curvature-based method) and even to full-fledged structure comparison programs. In the database search setting, CURVE can always recognize the query's structure family. For all example structures we tested, the top scoring hits returned by CURVE are indeed from the same families.

### Benchmarking against existing structure alignment programs

**Figure 3:**
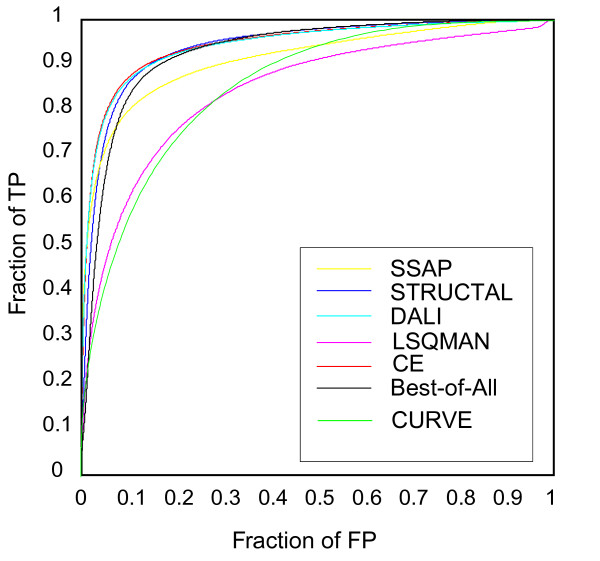
**Benchmark of CURVE against other structure alignment programs**. The ROC curves for other structure alignment programs are adapted from Figure 1a of Kolodny et al, 2005 [26].

Since our goal was not to produce yet another structure alignment program, but to explore the minimalistic definition of protein structures that can be used for structural comparisons, we did not optimize the alignment in terms of structure superposition, or develop a robust P-value scheme. Nonetheless, we compare the performance of the CURVE program with existing structure alignment programs in a benchmarking test using the setup of Kolodny *et al *[26]. In this test, 2930 representative protein structures are selected from diverse CATH superfamilies, and among all 8,581,970 pairs of structures, positive are defined as the pair of structures consisting of proteins from a same CATH topology category, and negative otherwise. For each structure pair (*i*,*j*) in the benchmark set, we define the native score of CURVE to be their dynamic programming score S(*i*,*j*) normalized by the self alignment scores, i.e., S_native_(*i*,*j*) = S(*i*,*j*)^2^/(S(*i*,*i*)S(*j*,*j*)). The ROC plot of CURVE (Figure 3) is shown together with the ROC plots for other structure alignment programs as described in [26]. Overall, CURVE's performance is comparable to LSQMAN [27]. At the regime of lower false positive, CURVE does not perform as well as LSQMAN, probably due to the backbone smoothing. At the regime of higher false positive, CURVE clearly outperforms LSQMAN, and even SSAP [28].

We also like to point out that a major advantage of CURVE is its speed. Theoretically the CURVE algorithm resembles the standard Smith-Waterman algorithm, with the time complexity O(*nm*), where *n *and *m *are the lengths of the two backbone chains to be compared. In our benchmarking test, CURVE took a total of 4629 CPU hours on a cluster with Pentium III 1 GHz CPUs. This result is comparable to LSQMAN, which was reported taking 1790 hours over 2.6 GHz CPUs (Table 5 of [26]). If we consider that CURVE is currently implemented as a prototype using Perl, and typically a reimplementation using C/C++ would speed up dramatically from Perl, this result suggests that CURVE can be used as a tool for fast filtration of potential hits before the application of more time-consuming structure alignment programs.

### Evaluating structure predictions

**Figure 4:**
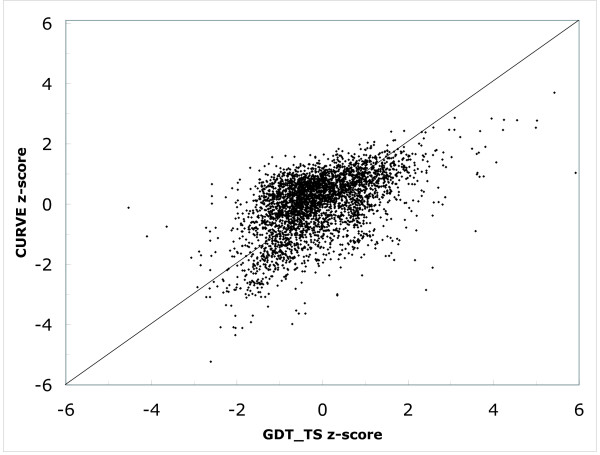
**Scatter plot of z-scores for CURVE versus GDT_TS over CASP6 targets**. The plot contains the comparison for all domains in the FR category except T0243, whose PDB code is not available on the CASP6 web site. The diagonal line *y *= *x *is drawn for aiding visual inspection and is not a regression fit.

In this test, we assume a CASP-like setting and use CURVE to rank predicted structures against the true target. We compare CURVE scores against the Global Distance Test Total Score (GDT_TS) [29]. We compare all fold recognition targets with their first predicted models. Following the setting in [30], we rescale the GDT_TS scores and CURVE score using the z-value among predictions for individual targets, and make a scatter plot (Figure 4). The CURVE score is in a good overall agreement with the GDT_TS measure. The two z-scores for 96% of the predictions differ by less than 2. Interestingly, we like to point out that there is no simple linear relation between the two z-scores. This result again illustrates that CURVE optimizes a different objective function than traditional RMSD-based structure superposition algorithms.

Notably, the CURVE score and the GDT_TS measure of some predicted structures do not agree: they either have a high CURVE and low GDT_TS score, or vice versa. We examine both types of disagreements. We find that predictions with a large CURVE score but a low GDT_TS measure, such T0251TS122_1, correspond to a prediction where the fold has been predicted correctly but the predicted secondary structure elements are shifted. On the other hand, predictions with a good GDT_TS measure but a low CURVE score, such T0251AL164_1, are models that are not be able to predict the overall topology. Nonetheless, we recognize that the evaluation of structure prediction is a subjective and difficult task. Our test demonstrates that CURVE provides a complementary perspective for such evaluations to that defined by RMSD-based criteria.

### Describing differences among NMR conformers

**Figure 5:**
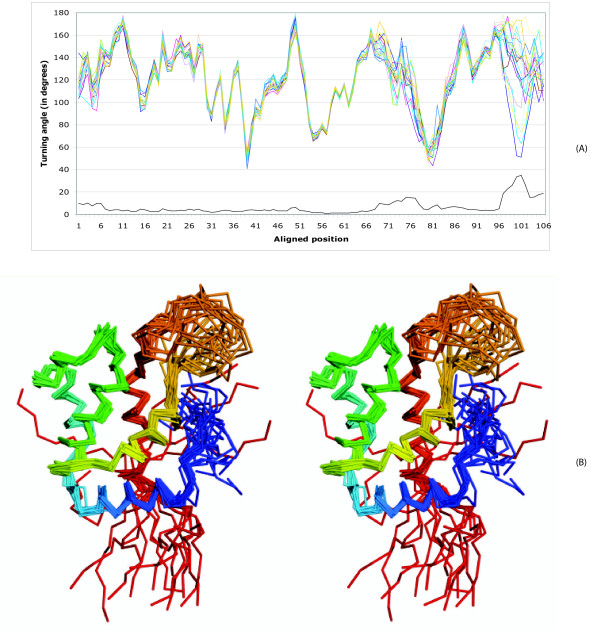
**Turning angle curves (A) of 20 NMR conformers (B) of 1tuz**. The line at bottom of (A) represents the standard deviations of turning angles at individual residues. (B) Stereo diagram of the structural superposition of these 20 conformers.

Different conformers of an NMR structure capture the flexibility of a protein in solution. In this experiment, we present an example to demonstrate the applicability of turning angle series in describing the conformation variability of a protein. We take the 20 conformers of an NMR structure of diacylglycerol kinase alpha protein, 1tuz, and overlay the turning angle curves of its conformers (Figure 5). We define the variability as the standard deviation of the turning angles at each residue. Three regions with variability are apparent: N-terminal, C-terminal, and residues 69–77. While the N-terminal region is only slightly variable, the C-terminal region is highly variable; in fact, it assumes two sub-structures (as seen in both structure superposition and turning angle overlap) causing a marked change in variability. The region at residues 69–77 corresponds to a loop between two α-helices.

### Recognizing similarity between drastically different conformations of the same protein

Some proteins assume drastically different structural conformations at different conditions (such as binding to different substrates) to fulfill their functions. The similarity between different structural conformations of a protein can go beyond what traditional RMSD-based structure alignment tools can recognize. Below we demonstrate that our method is particularly suited in identifying similarities between structures with such conformation changes.

**Figure 6:**
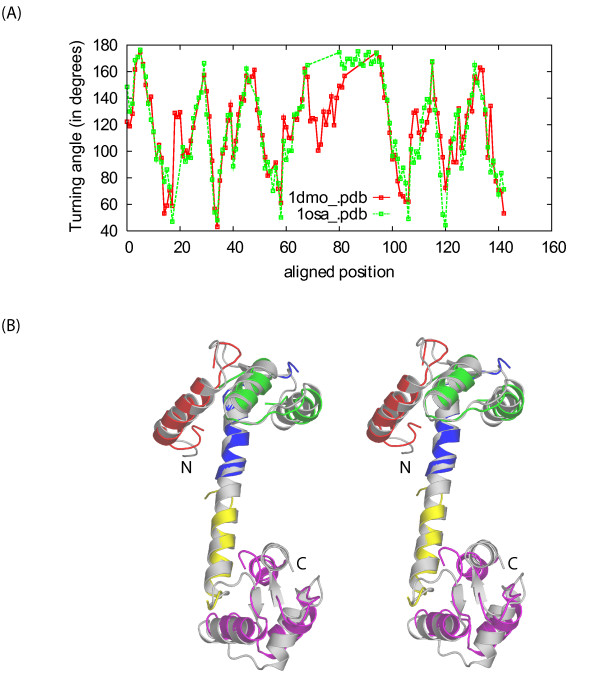
**Flexible alignment of different calmodulin structures: 1dmo (APO form) and 1osa (Ca-binding form)**. (A) Angle curve overlap graph of 1dmo and 1osa. (B) Stereo diagram of the structural superposition of 1dmo (colors) and 1osa (grey) generated by FATCAT. FATCAT breaks 1dmo into 5 rigid body segments (each segment is shown with a unique color) linked by hinges. Notably, the long α-helix in the middle of 1osa is broken into two smaller ones in 1dmo. See text for details. Higher gap penalties (opening:1000 and extension:333) were used so that the center region (alignment positions 65–95) appears to be "mismatch" instead of parallel gaps in both angle curves. This is only to enhance the presentation of the angle changes associated with the conformation change – the default parameters produce essentially the same result.

It is well known that the APO form and the calcium-binding form of calmodulin have distinct conformations [31]. In its calcium-binding form, calmodulin has two globular domains (N- and C- terminal domains) linked by a central α-helix. In its APO form, the central α-helix is broken into two short α-helices linked by a region with poorly defined secondary structure. In addition, the two globular domains experience some internal changes. Traditional structure alignment programs that are based on rigid body superposition can only align one of the terminal domains at a time and thus are unable to capture the overall conformation change. Angle series alignment produces a more realistic result. From Figure 6, it is clear that CURVE captures the conformation change breakage of the central α-helix with a region with large angle deviations, while the twists in both N-terminal and C-terminal domains only result in smaller changes in turning angle among few residues. The flexible alignment programs FATCAT [14] and FlexProt [15] can also align these two structures through their entire length; however, they have to introduce four hinges.

**Figure 7:**
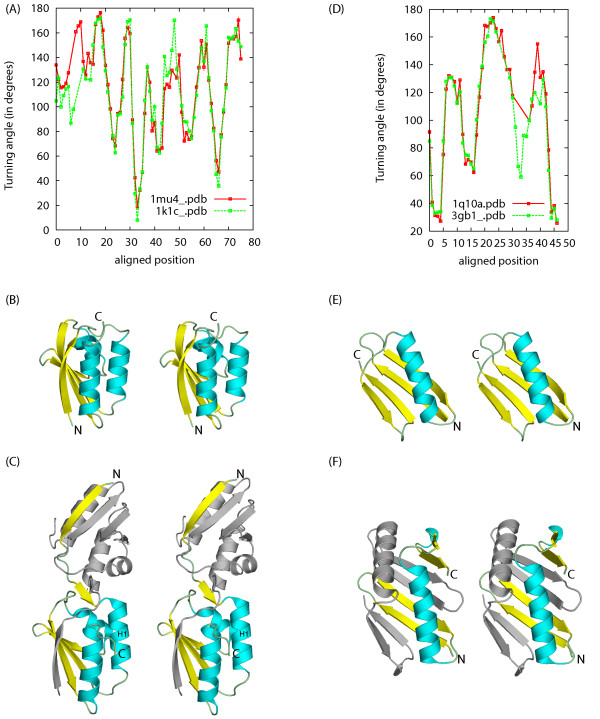
**Conformation changes when monomeric subunits form domain swapped oligomers**. Angle curve overlap graphs for 1mu4 and 1k1c:A (A) and for 1q10:A and 3gb1 (D); (B,C) stereo diagrams of 1mu4 and 1k1c:A with aligned regions highlighted in similar colors; (E,F) stereo diagrams of 1q10:A and 3gb1 with aligned regions highlighted in similar colors.

Conformation changes are also common when monomeric subunits form domain-swapped oligomers. We present two examples of such cases. In the first example, we compare two conformations of the catabolite repressor HPr-like protein from *Bacillus subtilis*. The monomer structure 1k1c:A has an anti-parallel β-strand of order 1423. In the dimer structure 1mu4, two subunits swap their N-terminal β-strands. The angle curve overlap graph of the CURVE result is shown in Figure 7. Similar to the case of aligning calmodulin structures, CURVE aligns both the main part (alignment positions 13–75) and the N-terminal swapped β-strand (alignment positions 1–7, shortened by smoothing at the N-terminus). Moreover, CURVE captures the subtle conformation change on the main part: notice the angle changes around the alignment positions 44–48.

In the second example we compare the immunoglobulin-binding domain B1 of streptococcal protein G (GB1), a favorite subject of studying protein folding and design. Mutants of GB1 are reported to adopt very different conformation from the wild type [32]. The wild type structure (PDBID: 3gb1) contains an α-helix and a four-stranded β-sheet made of two β-hairpins, one N-terminal and the other C-terminal to the α-helix. The structure of mutant HS#124F26A (PDBID: 1q10) reveals a domain-swapped dimer that involves exchange of the second β-hairpin. The resulting overall structure is comprised of an eight-stranded β-sheet whose concave side is covered by two α-helices. CURVE alignment reveals that the most significant angle change happens at the region between the α-helix and the second β-hairpin (Figure 7); all secondary structures remain mostly unchanged. In both cases, the conformation change results in structures which can align only with large RMSD, while the changes on turning angles are modest. In such cases, aligning structures by directly optimizing RMSD may not be a good choice. CURVE alignment directly captures the backbone turning angle changes associated with the conformational changes, which, we believe, is a better choice.

### Turning-angle profile model of a structure family

We have shown that the CURVE representation not only reveals similarity between structures, but also pinpoints the regions where they differ. We explore the generalization of CURVE representation to the modeling of multiple structure alignment of a protein family.

**Figure 8:**
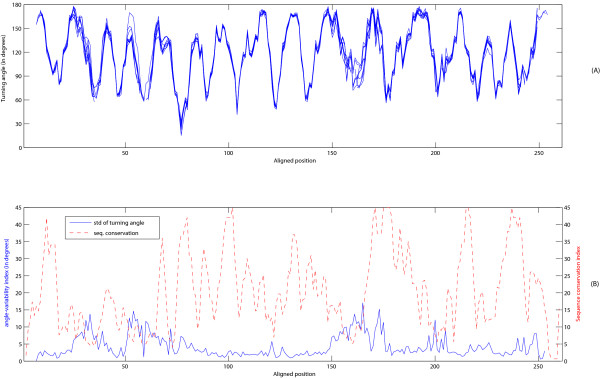
**Turning angle profiles modeling of 10 structures from the triosephosphate isomerase family**. (A) The turning angle profiles of 10 structures derived from the HOMSTRAD multiple alignment [33]. (B) The angle-variability index (standard deviation of turning angles) and sequence conservation index of their alignment are also shown.

We obtain multiple structure alignment of 10 structures of the triosephosphate isomerase family of the TIM-barrel topology from the expert-curated structure alignment database HOMSTRAD [33]. We overlay the turning angle profiles of these structures according to the HOMSTRAD alignment in Figure 8. In addition to the angle-variability index, we also calculate the sequence conservation index, defined as the number of matching pairs of amino acids in a given column in a multiple alignment. Not surprisingly, there is a negative correlation of the angle-variability index and the sequence conservation index.

The curve overlap graph in Figure 8 immediately suggests a profile representation of a structure family. One interesting direction for future research is to construct a profile HMM of curves for structures from a given protein family.

### Revealing similarities between structures from distinct folds but sharing structural (and often functional) similarities

**Figure 9:**
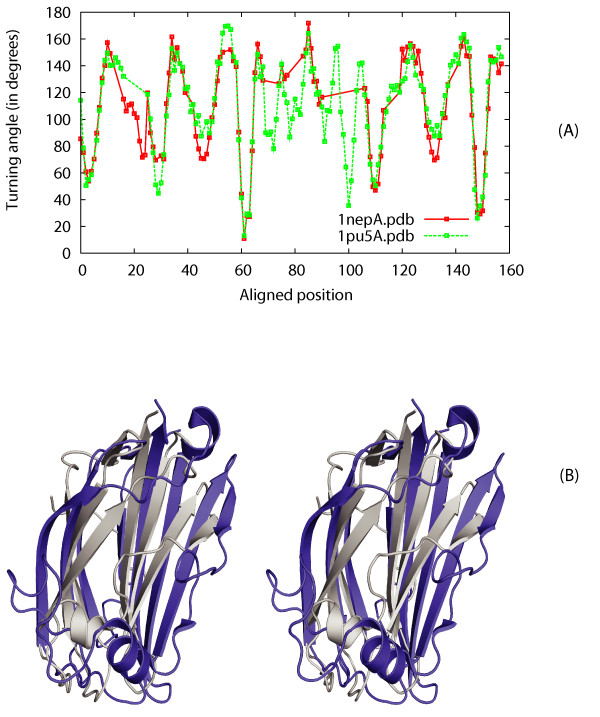
**Angle curve overlap of Ig-like β-sandwich fold structure 1nep:A and gm2 activator fold structure 1pu5:A**. (A) Angle curve overlap graph of 1nep:A and 1pu5:A. (B) Stereo diagram of the structural superposition of 1nep:A (colors) and 1pu5:A (grey).

We explore if CURVE alignment can detect similarities between structures from different SCOP folds that share similar functions. We report an interesting case showing the structural similarities between the Ganglioside M2 (gm2) activator fold and the immunoglobulin-like (Ig-like) β-sandwich fold. Similar to structures of the immunoglobulin-like fold, the gm2 activator structure 1pu5:A has two β-sheets with four β-strands each. However, the space between its two β-sheets is larger, serving as a lipid binding pocket. Thus, 1pu5:A is an "opened up" Ig-like fold. When comparing 1pu5:A against the Ig-like structure 1nep:A, CURVE found that both structures share a surprising similar turning angle curve (Figure 9). The two proteins are also seen to share sequence similarity as revealed by the profile-profile alignment and fold recognition program FFAS [34]. Arguably, CURVE reveals potential homology between the gm2 activator fold and the Ig-like β-sandwich fold.

## Discussion

The results presented in our manuscript bring up an interesting question: Since turning angle curve similarity is only a necessary, but not sufficient condition for structural similarity, why does CURVE alignment work so well? We postulate that this is because most natural proteins are constrained into a compact shape, and thus for a given turning angle series, there are only a small number of ways to arrange them into a realistic compact shape. For example, turning angle series cannot distinguish between right-handed and left-handed β/α/β units. Fortunately, right-handed β/α/β connections dominate over left-handed ones in naturally occurring proteins.

Our result also raises another interesting question on the structural constraints of protein evolution. For most structures, changes in their sequences caused by mutations such as substitutions and minor insertions or deletions only result in subtle changes in structure with the overall 3-D shape of the structure largely being preserved. However, for some structures, such as the GB1 protein, small mutations can result in a drastic change of their structural conformation and CURVE can be useful in detecting such changes.

The angle series alignment has some interesting implications. Traditional structure alignments have never been like sequence alignment. While sequence alignments typically define an edit distance, a score defined by a procedure via which one can transform one sequence into the other, existing structure alignment programs optimize RMSD of a superimposed subset of residues among structures. The result of such a structure alignment does not provide a series of operations that transform one structure into the other. Angle series alignment produces a set of angle matches that could be interpreted as a series of operations for structural transformation. Naively, one can bend every angle of one structure to the corresponding angle of the other structure. To derive a set of realistic backbone-bending operations, one needs to consider the stereochemical constraints of the backbone and correlation of the turning angles.

The current prototype implementation of angle series alignment certainly can be improved by incorporating additional information. For example, the alignment of angle curves can only give an alignment with a certain resolution due to the smoothing procedure. It is possible to implement an iterative refinement scheme which starts with an overall alignment of angle series based on a large smoothing radius, then iteratively refine the alignment by considering angle series based on smaller smoothing radii. Since angle series is only a "planar" feature, adding 3-D features such as handedness information will help (in cases where distinguishing between left and right is important).

## Conclusion

In this paper we introduce the turning angle series along smoothed backbone of the polypeptide as a new descriptor of protein structure. We demonstrate its utility in defining structural similarity by implementing and testing an alignment program, CURVE, based on this feature. Our results show that this simple approach works surprisingly well. Although not directly optimizing RMSD, the result of CURVE generally agrees with the SCOP structure classification and traditional structural alignment programs. Benchmarking results showed that CURVE's performance is comparable to popular structure alignment programs such as LSQMAN, while CURVE runs significantly faster. Moreover, CURVE can reveal similarities between drastically different conformations of the same protein structure, which is beyond the scope of traditional structure alignment programs. In aligning structures from different SCOP folds CURVE demonstrate its potential in identifying remote structural similarity.

## Methods

### Backbone smoothing and turning angles

Our backbone smoothing procedure follows that of [16]. We assign the center of gravity of every *k *consecutive C_α _atoms as a new pseudo-C_α _atom. With a proper choice of *k*, the resulting chain of pseudo-C_α _smoothes out the local "wiggles" due to the zigzags in β-strands or the spiral patterns in the α-helices and reveals the global fold of the protein structure as a smooth curve in 3-D space. Thus, we refer the chain of pseudo-C_α_'s as a *smoothed backbone *(Figure 1A). Our smoothing procedure suppresses the local high frequency curvature signals that arise from the local periodicity of the backbone, and thus reveals the overall topology of the structure.

We define the turning angle at each pseudo-C_α _atom along the smoothed backbone in order to reflect medium level topological features around it. Ideally, this turning angle should be close to 180° in the middle of a long straight segment along the smoothed backbone and small (close to 0°) at a sharp turn such as a β-hairpin. Also following the definition in [16], we define the turning angles at residue *i *as the angle between the two vectors [*i*-*d*+1,*i*-*d*] and [*i*+*d*-1,*i*+*d*]. The value of *d *determines the span of the angle definition, thus *d *is called the *angle defining distance*. Assuming that *d *is small, the fragment from residue *i*-*d *to *i*+*d *is almost planar and the torsional angles are negligible, this definition can be interpreted as the integral of the curvature function of the chain in this local interval.

We experimented with different choices of *d *(Figure 1B). Small values of *d *make all angles indistinctively large: they only capture local turns and are unable, for example, to describe the 180° turn in anti-parallel β-sheets. Large values of *d*, however, are uninformative for revealing local curvatures. Figure 1B demonstrates the effect of value *d *on the shape of the angle series curve: with decreasing *d *values, the first two plateaus in curve *d *= 1 dissolve into narrower and lower peaks, while the valley between them becomes deeper and wider. We choose *d *= 3 since it is the smallest value which gives a good dynamic range of angle values.

It is worth to note some general features of the angle series description of a protein structure. First, the plateaus and peaks (regions with high angle values) correspond to straight parts after smoothing, often long secondary structure elements or generalized secondary structure elements. For instance, in Figure 1B, the first plateau/peak corresponds to the first α-helix in structure and the second peak corresponds to the β-strand after the first α-helix). However, some straight segments do not correspond to classical secondary structure elements [16], we call such regions generalized secondary structure elements. Second, the valleys correspond to points where the path changes direction. Turning angle series is a rich description of the chain topology that also includes detailed turning characteristics, such as the length of the turn and the type of secondary structure (or generalized secondary structure) elements, with the latter described by the density of points along the smoothed chain [16].

In order to uniquely specify a path in three dimensions, both curvature and torsion angles would be required. The information about the torsion angles is lost in the representation of the path used here; therefore, it cannot distinguish whether the next straight element after a turn would be to the left or right of the original element. However, as we have shown in our study, using only the curvature angle series, we can still recognize most cases of the structural similarity between actual protein structures.

### Aligning turning angle series

We treat the turning angle series as a sequence of numbers. A natural way to compare such sequences is via dynamic programming. Protein and DNA sequences are described by discrete alphabet and could be aligned by the well known dynamic programming algorithms ([18,19]). The alignment of sequences of continuous numbers is rarely used in bioinformatics; however, it is very well studied in computer science as the time warp problem. Essentially, given two series indexed by time, the objective of time warp is to find the optimal matching between the points along the two time series. Typically, mismatches are penalized by the squared deviation of two time points (see [35] for a review).

In this study, we employ a standard time warp setting. Given two turning angle series (*a*_*i*_) and (*b*_*j*_), the goal is to find a maximally scoring gapped local alignment between them. The total score is the sum of scores of matching turning angle pairs with affine gap penalties. We adopt the standard affine gap penalty scheme. And we define the score for matching a pair of angles *a*_*i *_and *b*_*j *_as of the form -(*a*_*i *_- *b*_*j*_)^2^, i.e., the penalty of aligning two angle values increases quadratically with their angle difference. To avoid over-penalizing a large angle difference, the score has a lower cap. If all matching scores were negative, the optimal alignment would be of zero length. To encourage longer alignments, the matching score is augmented by a default reward *r*_0_. Any angle difference smaller than *r*_0 _is rewarded, otherwise it is penalized. Thus, the overall score for matching a pair of angles *a*_*i *_and *b*_*j *_is:

*S*(*a*_*i*_, *b*_*j*_) = *r*_0 _^2 ^- min [(*a*_*i *_- *b*_*j*_)^2^, (1.5*r*_0_)^2^].

Although our simplistic scoring scheme may produce unrealistic alignments (such as creating large gaps in the middle of secondary structure elements), we find that this scheme produces overall structure alignments that while not accurate enough for comparative structure modeling, are yet good enough to discover the overall structural similarity.

*r*_0 _and the gap opening and extension penalties are adjustable parameters. Based on parameter-tuning tests (data not shown), we found the alignment is not very sensitive to the choices of *r*_0 _and gap penalties as long as the alignment is in the log-phase [36]. In our experiments we choose default parameters to be *r*_0 _= 21 and gap opening/extension penalties 300/100. All these procedures are implemented as a program CURVE, available both via a webserver [37] and as a supplementary file (Additional file 1).

## Authors' contributions

DGZ conceived and implemented the idea of aligning curves along the smoothed backbone and analyzed the results. SSK contributed his expertise in structural similarity and analyzed the results. HC helped analyze the results. PP and AG are senior authors who conceived the overall idea, coordinated the collaboration and guided the progress of this study. DGZ, SSK, and AG drafted the paper.

## Supplementary Material

Additional File 1 A tarball file containing an implementation of the CURVE program and also programs that generate turning angle curve series. Click here for file
